# Clinical Impact of Staple-Line Oversewing in Totally Mechanical Collard Cervical Anastomosis for Esophageal Cancer

**DOI:** 10.3390/cancers18101513

**Published:** 2026-05-08

**Authors:** Koshiro Ishiyama, Ryoko Nozaki, Ryota Kakuta, Shota Igaue, Eigo Akimoto, Daichi Utsunomiya, Daisuke Kurita, Yasuyuki Seto, Hiroyuki Daiko

**Affiliations:** Division of Esophageal Surgery, National Cancer Center Hospital, 5-1-1 Tsukiji, Chuo-Ku, Tokyo 104-0045, Japan; koishiya@ncc.go.jp (K.I.); rynozak@ncc.go.jp (R.N.); rkakuta@ncc.go.jp (R.K.); sigaue@ncc.go.jp (S.I.); eiakimot@ncc.go.jp (E.A.); dutsunom@ncc.go.jp (D.U.); dakurit@ncc.go.jp (D.K.); yaseto@ncc.go.jp (Y.S.)

**Keywords:** esophageal cancer, esophagectomy, cervical esophagogastric anastomosis, anastomotic leakage, staple-line oversewing, totally mechanical Collard anastomosis, surgical outcomes

## Abstract

Background: Cervical esophagogastric anastomosis using a gastric conduit is commonly performed after esophagectomy for esophageal cancer, but anastomotic leakage remains a major postoperative complication. Totally mechanical Collard (TMC) anastomosis provides favorable stricture outcomes; however, leakage rates remain variable. The clinical benefit of staple-line oversewing in TMC anastomosis has not been fully clarified. Methods: This retrospective cohort study included consecutive patients who underwent esophagectomy with TMC cervical anastomosis between January 2017 and December 2024. Patients were divided into oversewing and non-oversewing groups according to whether staple-line oversewing was performed. The primary endpoint was anastomotic leakage. Secondary endpoints included leakage severity, timing of leakage onset, healing duration, anastomotic stricture, and postoperative complications. Multivariable logistic regression analysis was used to identify independent risk factors for leakage. Results: A total of 803 patients were included (oversewing: *n* = 313; non-oversewing: *n* = 490). Baseline characteristics were well balanced between the two groups. The incidence of anastomotic leakage was significantly lower in the oversewing group than in the non-oversewing group (4.4% vs. 8.1%, *p* = 0.043). Leakage severity was also reduced, with Grade I leakage occurring more frequently in the oversewing group (78.5% vs. 30%, *p* = 0.004). Leakage occurred later in the oversewing group (12.7 ± 7.6 vs. 8.9 ± 4.2 days, *p* = 0.01), whereas healing duration was comparable between groups. The incidence of anastomotic stricture did not differ significantly between the two groups (3.5% vs. 5.3%, *p* = 0.3). Multivariable analysis identified body mass index ≥25 kg/m^2^ (OR 2.37, 95% CI 1.08–4.93, *p* = 0.03) and the absence of staple-line oversewing (OR 2.15, 95% CI 1.03–4.82, *p* = 0.04) as independent risk factors for leakage. Conclusions: Staple-line oversewing of TMC cervical anastomosis was associated with a reduced incidence and milder severity of anastomotic leakage without increasing anastomotic stricture. This simple and reproducible technique may improve anastomotic stability after esophagectomy for esophageal cancer.

## Simple Summary

Esophagectomy for esophageal cancer is associated with a substantial risk of postoperative complications, among which cervical anastomotic leakage remains one of the most serious. Totally mechanical Collard (TMC) cervical anastomosis is widely used because of its favorable stricture profile; however, the clinical benefit of staple-line oversewing remains unclear. In this study, we evaluated whether additional seromuscular reinforcing sutures could reduce the risk and severity of anastomotic leakage. We analyzed more than 800 patients who underwent esophagectomy with TMC cervical anastomosis at a high-volume cancer center. Staple-line oversewing was associated with a lower incidence of anastomotic leakage, and when leakage occurred, it tended to be less severe. These findings suggest that simple staple-line oversewing may improve the safety and stability of cervical esophagogastric anastomosis after esophagectomy.

## 1. Introduction

Cervical anastomosis using a gastric conduit is commonly performed for reconstruction following esophagectomy in patients with esophageal cancer. Although advances in perioperative care and minimally invasive approaches have improved short-term outcomes, postoperative morbidity remains substantial [1,2]. Among the associated complications, anastomotic leakage (AL) is of particular concern because it can lead to prolonged hospitalization, infectious complications, and increased postoperative mortality. Recent multicenter analyses have shown that AL is one of the most frequent complications contributing directly to in-hospital death after esophagectomy, highlighting the critical importance of preventing this event [3].

Cervical esophagogastric anastomosis is widely performed as the standard reconstruction method following esophagectomy with three-field lymph node dissection for thoracic esophageal cancer [4,5]. Despite its widespread adoption, cervical AL continues to occur in 10–30% of patients and remains a major postoperative challenge [6,7,8]. Various anastomotic techniques—hand-sewn, circular stapled, and linear stapled—have been compared to reduce the risk of leakage and stricture, but no consensus exists regarding the optimal method [8,9]. Linear-stapled side-to-side techniques derived from the Collard et al. [10] have gained attention because of their wider anastomotic lumen and favorable stricture profile. Several studies from Japan and China have shown that modified Collard or Totally Mechanical Collard (TMC) anastomoses reduce stricture rates to approximately 2–10%, whereas reported leakage rates vary widely from 3% to 22% [11,12,13,14,15,16,17]. These discrepancies may be partly attributable to differences among studies in the type of staplers used and whether staple-line oversewing was applied during the TMC anastomosis.

Staple-line oversewing anastomoses has therefore attracted increasing interest as a potential strategy to mitigate anastomotic failure. In studies of circular-stapled esophagogastric anastomosis, staple-line oversewing has been reported to reduce the incidence of AL without increasing the risk of stricture or other adverse events [18]. However, such evidence is largely confined to circular-stapled or hand-sewn techniques, and its applicability to linear-stapled cervical anastomoses remains unclear. In the context of TMC anastomosis, the potential benefit of oversewing has not been adequately evaluated. In 2021, our institution introduced routine staple-line oversewing of the TMC cervical anastomosis with the aim of enhancing staple-line stability and reducing early mechanical disruption.

The present study aimed to compare postoperative outcomes between Oversewing and Non-oversewing TMC cervical anastomosis in a large consecutive cohort of patients undergoing esophagectomy for esophageal cancer. Specifically, we evaluated the incidence and severity of anastomotic leakage, as well as the timing of leakage onset and healing. Through this analysis, we sought to clarify the clinical value of staple-line oversewing TMC cervical anastomosis.

## 2. Materials and Methods

### 2.1. Study Design and Patient Selection

**Figure 1:**
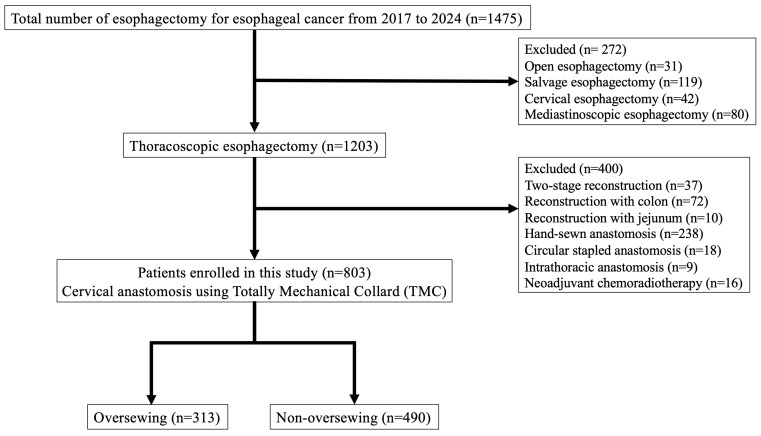
Study flow diagram of patient selection.

This retrospective cohort study included consecutive patients who underwent esophagectomy with cervical esophagogastric anastomosis at the National Cancer Center Hospital between January 2017 and December 2024. A total of 1475 esophagectomies were performed during the study period. Patients were excluded if they underwent open esophagectomy, salvage surgery, cervical esophagectomy, mediastinoscopic esophagectomy, two-stage reconstruction, reconstruction using organs other than the gastric conduit, or non-TMC anastomosis (hand-sewn or circular-stapled). After applying these criteria, total of 803 patients were included. The process of patient selection is summarized in Figure 1. According to whether staple-line oversewing was performed, patients were categorized into an oversewing group (*n* = 313) and a non-oversewing group (*n* = 490). From 2017 to 2020, non-oversewing TMC was routinely performed, whereas from 2021 onward, staple-line oversewing was added. Clinical data were extracted from institutional databases and electronic medical records.

### 2.2. Surgical Procedure

Esophagectomy was performed via thoracoscopic or robot-assisted approaches with two- or three-field lymphadenectomy according to tumor location and clinical stage.

After gastric mobilization, a gastric conduit approximately 4 cm in width was created along the greater curvature. The conduit was elevated to the neck through the posterior mediastinal or retrosternal route. Feeding jejunostomy was not routinely performed. A nasogastric feeding tube was placed intraoperatively.

### 2.3. Anastomotic Technique (TMC and Oversewing Modification)

**Figure 2:**
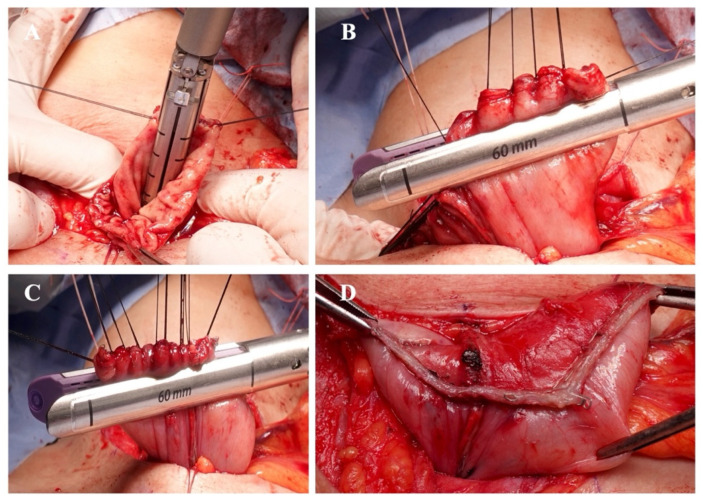
Surgical steps of totally mechanical Collard (TMC) cervical esophagogastric anastomosis. (**A**) Stapling of the posterior wall of the esophagus and gastric conduit using a 45 mm triple-row linear stapler. (**B**) First firing of a 60 mm linear stapler to close the anterior wall of the anastomosis. (**C**) Second firing of a 60 mm linear stapler to complete closure of the anterior wall. (**D**) Completed cervical esophagogastric anastomosis after stapling.

The TMC anastomosis used in this study was based on our previously reported technique [14]. It consists of a side-to-side anastomosis between the cervical esophagus and the gastric conduit. The posterior wall of the anastomosis was constructed using a 45 mm triple-row linear stapler (Tri-Staple™ purple cartridge, Medtronic, Minneapolis, MN, USA) (Figure 2A). After temporary closing of the anterior opening, the anterior wall was completed using two firings of a 60 mm linear stapler, resulting in a fully stapled anastomosis (non-oversewing technique) (Figure 2B–D).

**Figure 3:**
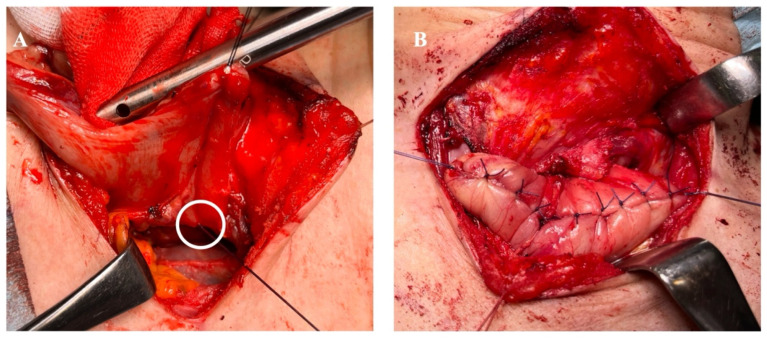
Staple-line oversewing during totally mechanical Collard (TMC) cervical esophagogastric anastomosis. (**A**) Seromuscular oversewing at the posterior corner of the anastomosis (white circle). (**B**) Completed staple-line oversewing of the anterior wall.

In the oversewing group, additional interrupted seromuscular sutures were placed to invert and reinforce the posterior edge of the anastomosis (Figure 3A) and the anterior staple line (Figure 3B) after completion of the TMC anastomosis. Reinforcing sutures were placed using 4-0 Vicryl (Ethicon, Somerville, NJ, USA). All cervical anastomoses were performed by attending staff esophageal surgeons using a standardized institutional technique.

### 2.4. Postoperative Management

Enteral nutrition was initiated via the nasogastric feeding tube on postoperative day (POD) 1 at 10 mL/day and gradually increased to 60 mL/day. Routine contrast esophagography was performed on POD 7. If no signs of leakage were observed, oral intake was initiated. If clinical signs suggestive of anastomotic leakage (e.g., cervical swelling or erythema) were present before POD 7, additional imaging was performed at that time.

### 2.5. Outcome Measures

The primary endpoint was the incidence of AL. Secondary endpoints included leakage severity, time to leakage onset, duration from leakage diagnosis to healing, anastomotic stricture, other postoperative complications, and mortality. The median follow-up was 36.9 months overall, and 22.4 and 54.8 months in the oversewing and non-oversewing groups, respectively.

### 2.6. Definitions

Anastomotic leakage was defined as clinical signs of cervical infection, such as swelling or erythema, with radiologic confirmation of contrast extravasation on esophagography or computed tomography.

**Figure 4:**
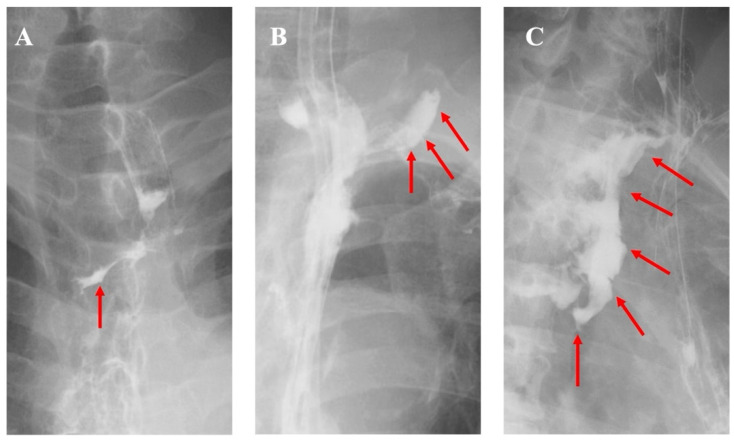
Radiographic grading of anastomotic leakage. (**A**) Grade I: linear contrast leakage without cavity formation. (**B**) Grade II: localized cavity near the anastomosis. (**C**) Grade III: leakage extending into the mediastinum or thoracic cavity. Red arrows indicate extraluminal leakage of contrast medium.

Leakage was graded according to esophagogram findings, as previously described [19] and illustrated in Figure 4. Leak severity was classified into three grades: Grade I, linear contrast extravasation without cavity formation; Grade II, formation of a localized cavity adjacent to the anastomosis; and Grade III, extension of the leak into the mediastinum or thoracic cavity.

Anastomotic stricture was defined as dysphagia requiring endoscopic intervention and inability of a standard endoscope to pass through the anastomosis in the absence of tumor recurrence. Refractory stricture was defined as the need for five or more endoscopic dilations. Other postoperative complications were defined as Clavien–Dindo [20] grade II or higher. The incidence of postoperative complications was defined as the proportion of patients experiencing at least one complication. Tumor staging was determined according to the UICC TNM classification, 7th edition [21].

### 2.7. Management of Anastomotic Leakage

Treatment strategy was determined according to leak grade, as previously described [19]. Patients with Grade I leaks were managed conservatively with fasting and nasogastric decompression. Patients with Grade II or III leaks underwent percutaneous trans-anastomotic drainage (PTD) whenever feasible. In cases of Grade III leakage with mediastinal or thoracic extension, additional transcervical or transthoracic drainage was performed when necessary. PTD was performed under radiologic guidance. A 14-Fr sump drainage tube (Cardinal Health, Dublin, OH, USA) was inserted through the cervical wound and advanced through the leak site into the gastric conduit. The distal side holes of the tube were positioned to straddle the leak site, allowing simultaneous drainage of the conduit and abscess cavity. Intermittent negative pressure of −99 cmH_2_O was applied to promote effective drainage and facilitate integration of the abscess cavity into a linear fistula. When the cavity size decreased, the nasogastric decompression tube was removed, and enteral feeding was continued via a cervical route for patient comfort. Oral intake was resumed after radiologic confirmation of leak closure. The leakage management protocol remained consistent throughout the study period.

### 2.8. Statistical Analysis

Continuous variables were expressed as mean ± standard deviation and compared using Student’s *t*-test or the Mann–Whitney U test as appropriate. Categorical variables were compared using the chi-square or Fisher’s exact test. Multivariable logistic regression analysis was performed to identify independent risk factors for anastomotic leakage. Odds ratios with 95% confidence intervals were calculated. Covariates included in the multivariable model were selected a priori based on clinical relevance and previous literature on risk factors for anastomotic leakage, including smoking status, body mass index, pulmonary function, intraoperative blood loss, and staple-line oversewing. All statistical analyses were conducted using JMP version 14 (SAS Institute Inc., Cary, NC, USA). A two-sided *p* value < 0.05 was considered statistically significant.

### 2.9. Use of Generative Artificial Intelligence

Generative artificial intelligence (ChatGPT-5.4, OpenAI, San Francisco, CA, USA) was used to assist with drafting and language editing of parts of the manuscript. The tool was used solely for language assistance and did not influence the study design, data collection, data analysis, interpretation of the data, or conclusions of the study. All outputs were critically reviewed and revised by the authors, who take full responsibility for the content of the manuscript.

## 3. Results

### 3.1. Patient Characteristics

Baseline patient characteristics are summarized in Table 1. There were no significant differences between the two groups in age (65.7 ± 9.6 vs. 64.9 ± 9.2 years, *p* = 0.236) or sex distribution (male: 76.1% vs. 80.4%, *p* = 0.157). Body mass index, skeletal muscle mass index, and handgrip strength were comparable between the groups. Performance status and the prevalence of comorbidities were similar. Preoperative laboratory parameters, including hemoglobin, albumin, prognostic nutritional index, and HbA1c, showed no significant differences. Pulmonary function, assessed by vital capacity and forced expiratory volume in 1 s, was also comparable between groups. Regarding tumor characteristics, tumor location, histologic type, receipt of neoadjuvant chemotherapy, and clinical stage distribution did not differ significantly between the groups. Overall, baseline characteristics were well balanced between the two groups.

### 3.2. Operative Outcomes

Operative outcomes are summarized in Table 2. The proportion of robot-assisted procedures was significantly higher in the oversewing group than in the non-oversewing group (51.4% vs. 28.2%, *p* < 0.001). Accordingly, total operative time was longer in the oversewing group (394 ± 70 vs. 354 ± 74 min, *p* < 0.001), as was thoracic operative time (162 ± 52 vs. 149 ± 54 min, *p* = 0.008). Estimated blood loss and the rate of intraoperative blood transfusion did not differ significantly between groups. Reconstruction route and the extent of lymph node dissection were comparable. The number of harvested lymph nodes, including total and thoracic lymph node yield, was similar between groups. Although R0 resection rates tended to be higher in the oversewing group, the difference did not reach statistical significance.

### 3.3. Postoperative Outcomes

Postoperative outcomes are summarized in Table 3. The incidence of anastomotic leakage was significantly lower in the oversewing group than in the non-oversewing group (4.4% vs. 8.1%, *p* = 0.043). The absolute risk reduction was 3.7%, corresponding to a number needed to treat of 27. In contrast, the incidence of anastomotic stricture did not differ significantly between groups, either overall (3.5% vs. 5.3%, *p* = 0.3) or for severe stricture (2.5% vs. 3.2%, *p* = 0.71). The oversewing group had lower rates of pneumonia (13.1% vs. 21.8%, *p* = 0.002) and vocal cord paralysis (7.9% vs. 12.8%, *p* = 0.036). Wound infection was also less frequent in the oversewing group (2.5% vs. 6.9%, *p* = 0.005). Overall, postoperative complications occurred less frequently in the oversewing group (57.1% vs. 70.6%, *p* < 0.001), as did severe complications (16.6% vs. 25.3%, *p* = 0.003). There were no significant differences in arrhythmia, chylothorax, reoperation rate, 90-day mortality, intensive care unit stay, postoperative hospital stay, or 30-day readmission between the two groups.

### 3.4. Risk Factors for Anastomotic Leakage

**Figure 5:**
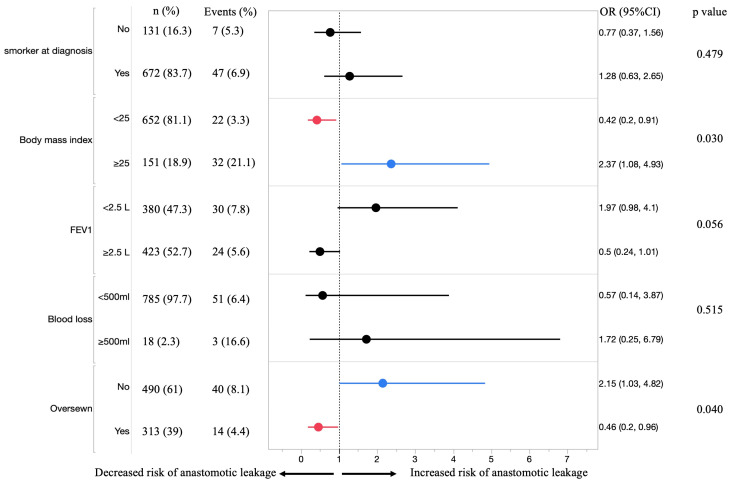
Multivariable logistic regression analysis of risk factors for anastomotic leakage. Forest plot showing odds ratios (ORs) and 95% confidence intervals (CIs) for factors associated with anastomotic leakage.

Multivariable logistic regression analysis identified two independent factors associated with a risk of anastomotic leakage (Figure 5). Body mass index ≥25 kg/m^2^ was associated with an increased risk of leakage (OR 2.37, 95% CI 1.08–4.93, *p* = 0.03). Likewise, the absence of staple-line oversewing was independently associated with a higher risk of leakage (OR 2.15, 95% CI 1.03–4.82, *p* = 0.04). Other examined variables were not significantly associated with leakage after adjustment.

### 3.5. Severity and Clinical Course of Anastomotic Leakage

**Figure 6:**
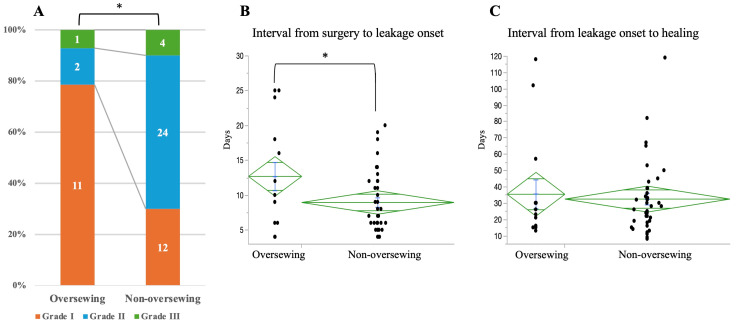
Comparison of leakage severity and clinical course between the oversewing and non-oversewing groups among patients with anastomotic leakage. (**A**) Distribution of leakage severity grades. (**B**) Time from surgery to onset of leakage. (**C**) Time from leakage diagnosis to healing. * indicates *p* < 0.05.

Among patients who developed anastomotic leakage (oversewing group: *n* = 14; non-oversewing group: *n* = 40), leakage severity differed between groups (Figure 6A). Grade I leakage was significantly more frequent in the oversewing group than in the non-oversewing group (78.5% vs. 30%, *p* = 0.004). The interval from surgery to leakage onset was longer in the oversewing group (12.7 ± 7.6 vs. 8.9 ± 4.2 days, *p* = 0.01) (Figure 6B). In contrast, the duration from leakage diagnosis to healing did not differ significantly between groups (35.5 ± 33.6 vs. 32.5 ± 21.3 days, *p* = 0.75) (Figure 6C).

## 4. Discussion

This study demonstrated that staple-line oversewing of the TMC cervical anastomosis was associated with a significantly lower incidence of anastomotic leakage compared with the non-oversewing technique. The protective effect of oversewing remained significant in multivariable analysis, and the severity of leakage was also reduced, with Grade I leakage predominating in the oversewing group. In addition, staple-line oversewing delayed the onset of leakage, whereas the duration required for healing was similar between the two groups. To our knowledge, this study is the first to directly compare clinical outcomes between oversewing and non-oversewing TMC cervical anastomoses.

The TMC techniques have gained widespread acceptance due to their favorable structure profile compared with circular stapled or hand-sewn anastomoses [8,9]. However, reported leakage rates for TMC anastomosis vary considerably across institutions [11,12,13,14,15,16,17]. Differences in stapling devices, conduit configuration, and perioperative management may partially account for this variability. Reinforcement of the esophagogastric anastomosis has also been investigated in circular stapled techniques. A randomized controlled trial evaluating fibrin sealant for reinforcement did not demonstrate a reduction in anastomotic leakage [22], whereas another study reported reduced leakage by burying the circular stapled anastomosis with additional sutures [18].

In addition, omental flap wrapping around the esophagogastric anastomosis has been reported as another strategy to reduce leakage [23]. However, the condition and volume of the omentum vary substantially among patients, and a bulky omentum may occasionally interfere with gastric conduit elevation. In contrast, seromuscular oversewing sutures can be applied easily and consistently without altering conduit configuration, which may provide a more reproducible reinforcement strategy. Furthermore, this technique does not require additional materials or devices and can be readily incorporated into routine surgical practice. The present study therefore specifically assessed the clinical impact of staple-line oversewing in TMC cervical anastomosis.

The reduction in leakage observed in the present study may be explained by improved mechanical stability at the staple line. Linear-stapled side-to-side anastomoses create a relatively wide lumen but expose the anterior and posterior staple lines to tension generated during conduit elevation and early postoperative swallowing. Additional interrupted seromuscular sutures may distribute tension more evenly and reinforce the anastomotic edge, thereby reducing focal stress at the staple line. This mechanical effect is supported by the observation that leakage severity differed between groups. In the oversewing group, Grade I leakage was significantly more frequent, whereas more extensive cavity formation was less common. These findings suggest that reinforcement may limit the extent of disruption even when leakage occurs.

The timing of leakage onset also differed between the groups. Leakage was diagnosed later in the oversewing group compared with the non-oversewing group. This finding may suggest improved early mechanical stability of the anastomosis. However, it should be interpreted with caution, as differences in surveillance timing and detection thresholds may have influenced this observation. In our protocol, routine contrast esophagography was performed on POD 7, with additional imaging triggered by clinical suspicion. Therefore, earlier detection in symptomatic patients cannot be excluded and may have affected the observed timing of leakage. From a mechanistic perspective, early leakage is generally considered to reflect primary mechanical failure of the anastomosis, whereas later leakage may be influenced more strongly by biological factors such as tissue perfusion and wound healing. Experimental studies of esophageal anastomotic leakage have suggested that the progression and resolution of leakage are closely associated with local inflammatory responses and tissue repair processes [24,25]. The delayed onset observed in the oversewing group therefore supports the hypothesis that reinforcement primarily improves the early mechanical integrity of the anastomosis. From a clinical perspective, the timing of leakage may also influence its severity. In the early postoperative period, leakage may allow digestive fluids to spread from the cervical wound into the mediastinum, potentially leading to severe mediastinitis. In contrast, when leakage occurs later in the postoperative course, adhesions around the anastomotic site may have already formed, which can limit the spread of digestive fluids into the mediastinum and thereby reduce the risk of severe infection. Consequently, even when leakage occurs, delayed onset may contribute to a less severe clinical course. Importantly, the duration from leakage diagnosis to healing did not differ significantly between the groups. Previous studies of circular stapled esophagogastric anastomosis have similarly suggested that the healing duration of anastomotic leakage may depend more on the characteristics and location of the leak than on the initial anastomotic technique [26]. These findings support the concept that reinforcement primarily affects the early mechanical phase of anastomotic failure rather than the subsequent biological healing process.

Body mass index ≥25 kg/m^2^ was identified as an independent risk factor for leakage in the present study. Patients with higher body mass index often have thicker cervical soft tissue, which may increase compression around the anastomotic site and contribute to impaired perfusion or increased tension. Consistent with our findings, previous meta-analyses have also reported higher body mass index as a risk factor for anastomotic leakage [27]. However, Anastomotic leakage is widely recognized as a multifactorial complication. Previous studies have shown that patient-related factors, operative factors, and gastric conduit perfusion—including conditions such as cardiovascular disease, diabetes, operative blood loss, and impaired conduit blood flow—may all contribute to its development [27,28,29,30,31]. These findings indicate that the development of anastomotic leakage likely results from the complex interaction of multiple factors rather than a single determinant.

A potential concern regarding staple-line oversewing is the risk of anastomotic stricture. However, the incidence of stricture in the present study was low and did not differ significantly between the groups. Similarly, previous studies evaluating oversewing in circular stapled esophagogastric anastomosis have reported that additional suturing reduces anastomotic leakage without increasing anastomotic stricture [18]. In our technique, oversewing sutures were placed in an interrupted seromuscular fashion without excessive tightening, thereby preserving luminal diameter and vascular supply.

Several limitations should be acknowledged. First, this was a retrospective study conducted at a single high-volume center. Second, the introduction of oversewing coincided with a later time period during which robot-assisted surgery became more prevalent. This temporal overlap introduces the potential for time-dependent confounding, including improvements in surgical experience, perioperative management, and postoperative care. Although perioperative protocols remained largely consistent during the study period, and key indicators of surgical quality, such as the R0 resection rate, and lymph node yield were comparable between groups, the influence of unmeasured confounders cannot be fully excluded. Routine contrast esophagography was performed in all patients on POD 7. Earlier imaging was performed in symptomatic cases, which may have introduced some degree of detection bias, particularly regarding the timing and severity of leakage. Propensity score–based methods such as matching or inverse probability weighting were not applied in this study. Although these approaches may further reduce confounding, baseline patient and tumor characteristics were well balanced between the groups, and the number of leakage events was relatively limited. Therefore, propensity score matching was expected to substantially reduce the effective sample size and compromise statistical power. While the cervical anastomosis was performed using a standardized technique irrespective of the thoracic approach, differences in surgical platform may still have indirectly influenced outcomes. However, previous studies comparing robot-assisted and conventional minimally invasive esophagectomy have reported comparable rates of anastomotic leakage, including both a randomized controlled trial and a multicenter propensity score–matched analysis. These findings suggest that the direct impact of thoracic approach on cervical anastomotic outcomes may be limited [32,33]. The median follow-up period differed between the groups (22.4 vs. 54.8 months), and shorter follow-up in the oversewing group may have underestimated late-onset strictures; therefore, these findings should be interpreted with caution. Third, long-term oncological outcomes were not evaluated. Despite these limitations, this study has several strengths. It included a large consecutive cohort treated under a consistent institutional protocol for anastomotic construction and leakage management. In addition, leakage severity and timing were systematically analyzed, providing detailed insight into the clinical course of anastomotic leakage.

## 5. Conclusions

Staple-line oversewing in TMC cervical anastomosis was associated with a lower incidence and milder severity of anastomotic leakage without increasing anastomotic stricture after esophagectomy for esophageal cancer. However, these findings should be interpreted cautiously because of the retrospective design and potential residual confounding. Therefore, this study should be considered hypothesis-supporting rather than definitive. A prospective multicenter registry may represent the most practical next step to further evaluate the clinical value and generalizability of this technique.

## Figures and Tables

**Table 1:** Patient characteristics.

Characteristic	Oversewing (*n* = 313)	Non-Oversewing (*n* = 490)	*p* Value
Age (years)	65.7 ± 9.6	64.9 ± 9.2	0.236
Sex			
Male	238 (76.1%)	394 (80.4%)	0.157
Female	75 (23.9%)	96 (19.6%)	
BMI (kg/m^2^)	22.4 ± 3.5	22.4 ± 3.3	0.951
SMI (kg/m^2^)	7.3 ± 1.1	7.5 ± 1.2	0.124
HGS (kg)	30.8 ± 7.7	29.5 ± 7.7	0.074
Smoker at diagnosis	84 (26.8%)	158 (32.2%)	0.232
Performance status			
0	285 (91.1%)	446 (91.0%)	0.838
1	26 (8.3%)	41 (8.4%)	
2	2 (0.6%)	2 (0.4%)	
3	0 (0%)	1 (0.2%)	
Comorbidities			
Cardiovascular	10 (3.1%)	8 (1.6%)	0.151
Cerebrovascular	13 (4.2%)	22 (4.5%)	0.861
COPD	9 (2.9%)	26 (5.3%)	0.112
DM	36 (11.5%)	66 (13.5%)	0.448
Renal	6 (1.9%)	15 (3.0%)	0.372
Chronic steroid use	3 (0.9%)	6 (1.2%)	0.749
Albumin (g/dL)	3.9 ± 0.3	4.0 ± 0.4	0.508
Hemoglobin (g/dL)	12.0 ± 1.6	12.3 ± 1.6	0.724
PNI	46.4 ± 4.9	47.4 ± 5.3	0.157
HbA1c (%)	5.6 ± 0.6	5.7 ± 0.6	0.323
VC (L)	3.4 ± 0.8	3.5 ± 0.7	0.062
FEV1 (L)	2.4 ± 0.6	2.6 ± 0.6	0.889
Tumor location			
Upper	32 (10.2%)	60 (12.2%)	0.525
Middle	159 (50.8%)	255 (52.1%)	
Lower	122 (39.0%)	175 (35.7%)	
Histologic type			
SCC	278 (88.8%)	422 (86.1%)	0.281
AC	35 (11.2%)	68 (13.9%)	
Neoadjuvant chemotherapy	253 (80.8%)	376 (76.7%)	0.187
Clinical stage			
I	74 (23.6%)	119 (24.3%)	0.892
II	65 (20.8%)	111 (22.7%)	
III	135 (43.1%)	204 (41.6%)	
IV	39 (12.5%)	56 (11.4%)	

Values are presented as *n* (%), or mean ± standard deviation, as appropriate. BMI, body mass index; SMI, skeletal muscle mass index; HGS, handgrip strength; COPD, chronic obstructive pulmonary disease; DM, diabetes mellitus; PNI, prognostic nutritional index; VC, vital capacity; FEV1, forced expiratory volume in 1 s; SCC, squamous cell carcinoma; AC, adenocarcinoma.

**Table 2:** Operative outcomes.

Characteristic	Oversewing (*n* = 313)	Non-Oversewing (*n* = 490)	*p* Value
Surgical approach			
Thoracoscopic	152 (48.6%)	352 (71.8%)	<0.001
Robot-assisted	161 (51.4%)	138 (28.2%)	
Operation time (min)			
Total	394 ± 70	354 ± 74	<0.001
Thoracic	162 ± 52	149 ± 54	0.008
Estimated blood loss (mL)	76 (48–126)	74 (45–137)	0.784
Intraoperative blood transfusion	13 (4.2%)	22 (4.5%)	0.861
Reconstruction route			
Retrosternal	297 (94.9%)	479 (97.8%)	0.085
Posterior mediastinal	15 (4.8%)	10 (2.0%)	
Antethoracic	1 (0.3%)	1 (0.2%)	
Field of lymph node dissection			
2 fields	42 (13.4%)	46 (9.4%)	0.082
3 fields	271 (86.6%)	444 (90.6%)	
Residual tumor			
R0	307 (98.2%)	463 (94.5%)	0.068
R1	3 (0.9%)	5 (1.0%)	
R2	3 (0.9%)	22 (4.5%)	
Lymph node yield			
Total	48 ± 13	48 ± 13	—
Thoracic	21 ± 6	21 ± 7	0.958

Values are presented as *n* (%), median (interquartile range), or mean ± standard deviation, as appropriate.

**Table 3:** Postoperative outcomes.

Characteristic	Oversewing (*n* = 313)	Non-Oversewing (*n* = 490)	*p* Value
Anastomotic leakage	14 (4.4%)	40 (8.1%)	0.043
Anastomotic stricture			
Overall	11 (3.5%)	26 (5.3%)	0.300
Severe	8 (2.5%)	16 (3.2%)	0.710
Pneumonia	41 (13.1%)	107 (21.8%)	0.002
Vocal cord paralysis	25 (7.9%)	63 (12.8%)	0.036
Arrhythmia	10 (3.1%)	25 (5.1%)	0.218
Reoperation	5 (1.6%)	5 (1.0%)	0.523
Wound infection	8 (2.5%)	34 (6.9%)	0.005
Chylothorax	5 (1.6%)	14 (2.8%)	0.342
Total complications	179 (57.1%)	346 (70.6%)	<0.001
Severe complications (grade ≥ III)	52 (16.6%)	124 (25.3%)	0.003
90-day mortality	2 (0.6%)	3 (0.6%)	1.000
ICU stay (days)	3 (2–3)	3 (2–3)	0.439
Postoperative hospital stay (days)	13 (11–17)	13.5 (11–17)	0.366
30-day readmission	14 (4.4%)	24 (4.9%)	0.865

Values are presented as *n* (%), or median (interquartile range), as appropriate. *p*-values were calculated using Fisher’s exact test where appropriate. ICU, intensive care unit.

## Data Availability

The data presented in this study are available within the article. Further inquiries can be directed to the corresponding author.
